# De novo transcriptome analysis for examination of the nutrition metabolic system related to the evolutionary process through which stick insects gain the ability of flight (Phasmatodea)

**DOI:** 10.1186/s13104-021-05600-0

**Published:** 2021-05-13

**Authors:** Takuma Sakamoto, Shunya Sasaki, Nobuki Yamaguchi, Miho Nakano, Hiroki Sato, Kikuo Iwabuchi, Hiroko Tabunoki, Richard J. Simpson, Hidemasa Bono

**Affiliations:** 1 Institute of Global Innovation Research, Tokyo University of Agriculture and Technology, 3-5-8 Saiwai-cho, Fuchu, Tokyo 183-8509 Japan; 2 Department of Science of Biological Production, Graduate School of Agriculture, Tokyo University of Agriculture and Technology, 3-5-8 Saiwai-cho, Fuchu, Tokyo 183-8509 Japan; 3 Department of Biochemistry and Genetics, La Trobe Institute for Molecular Science (LIMS), La Trobe University, Melbourne, VIC 3086 Australia; 4 Database Center for Life Science (DBCLS), Joint Support-Center for Data Science Research, Research Organization of Information and Systems (ROIS), Mishima, Shizuoka 411-8540 Japan; 5 Program of Biomedical Science, Graduate School of Integrated Sciences for Life, Hiroshima University, 3-10-23 Kagamiyama, Higashi-Hiroshima, Hiroshima 739-0046 Japan

**Keywords:** Stick insect, RNA sequencing, Transcriptome assembly, Transcriptome database, Enolase, Glycolytic pathway

## Abstract

**Objective:**

Insects are the most evolutionarily successful groups of organisms, and this success is largely due to their flight ability. Interestingly, some stick insects have lost their flight ability despite having wings. To elucidate the shift from wingless to flying forms during insect evolution, we compared the nutritional metabolism system among flight-winged, flightless-winged, and flightless-wingless stick insect groups.

**Results:**

Here, we report RNA sequencing of midgut transcriptome of *Entoria okinawaensis*, a prominent Japanese flightless-wingless stick insect, and the comparative analysis of its transcriptome in publicly available midgut transcriptomes obtained from seven stick insect species. A gene enrichment analysis for differentially expressed genes, including those obtained from winged vs wingless and flight vs flightless genes comparisons, revealed that carbohydrate metabolic process-related genes were highly expressed in the winged stick insect group. We also found that the expression of the mitochondrial enolase superfamily member 1 transcript was significantly higher in the winged stick insect group than in the wingless stick insect group. Our findings could indicate that carbohydrate metabolic processes are related to the evolutionary process through which stick insects gain the ability of flight.

**Supplementary Information:**

The online version contains supplementary material available at 10.1186/s13104-021-05600-0.

## Introduction

The evolutionary success of insects has been attributed to their flight ability and small size. Specifically, insects are able to expand to new environmental niches due to their flight ability [1–3]. More than 3000 species of stick insects exist worldwide, and some females have lost the ability of flight [4]. Interestingly, wingless insects tend to exhibit higher female fecundity [5]. In addition, some stick insects lost their flight ability despite having wings, and this feature might play an important role in evolutionary diversification [6]. The loss of flight ability could be involved in nutrient metabolism to produce energy, but the differences in the nutrient metabolic system between flight and flightless insects and between winged and wingless insects have not been examined.

The midgut is a main organ that contributes to food digestion and nutrient metabolism [7]. It has been suggested that the insect midgut physiology is strongly affected by the presence of unique cell types that confer peculiar features to this organ, and these midgut cells might support species-specific nutrient metabolism [8].

The stick insect *Entoria okinawaensis* has the largest body size among Japanese stick insect species and is wingless, which resulted in the loss of its flying ability. *E. okinawaensis* is both male and female and undergoes sexual reproduction [9]. We are interested in why stick insects lost their flight ability during the process of evolution and the relationship between nutrition metabolism and flight ability in stick insects.

In this study, we compared the nutrient metabolic systems of flight-winged, flightless-winged and flightless-wingless stick insect groups using our midgut transcriptome data and those from public database and found the expression of transcripts related to the production of energy in carbohydrate metabolic processes in the winged stick insect groups.

## Main text

### Methods

#### Insects

We obtained *Entoria okinawaensis* from Amami-Ohshima in Kagoshima, Japan, in 2011 and Ishigaki Island in Okinawa, Japan, in 2013 and maintained the insects in an insectary room at Tokyo University of Agriculture and Technology prior to collecting their eggs for this study. The eggs were maintained at 25 °C under 70% humidity and a 16-h light/8-h dark cycle. After hatching, the first-instar nymphs were maintained on young rose leaves, and the insects at the second-instar nymph to adult stages were maintained on *Quercus myrsinifolia* or rose (*Rosa multiflora*) leaves under 70% humidity and a 16-h light/8-h dark cycle.

*Sipyloidea sipylus* eggs were gifted by Dr. Takeshi Yokoyama at Tokyo University of Agriculture and Technology. The eggs were maintained at 25 °C under 70% humidity and a 16-h light/8-h dark cycle. After hatching, the first-instar nymphs were maintained on young rose leaves, and the insects from the second-instar nymph to the adult stage were maintained on rose (*Rosa multiflora*) leaves under 70% humidity and a 16-h light/8-h dark cycle.

### Sample collection and purification of total RNA

The midguts (n = 3; samples 1 and 2 were females, and sample 3 was male) and fat bodies (n = 3) were dissected from adult *E. okinawaensis*, and the midguts (n = 3) and fat bodies (n = 3) from adult *S. sipylus* were also dissected. These tissues were stored at − 80 °C until use. The midguts and fat body were weighed, homogenized with lysis buffer from a PureLink^®^ RNA extraction kit (Thermo Fisher Scientific Inc., Valencia, CA, USA) and then centrifuged at 13,000×*g* for 10 min. The supernatants were then collected and processed for RNA purification according to the manufacturer’s instructions. Purified total RNA (1 μg) samples were processed for RNA sequencing or quantitative RT-PCR (qRT-PCR).

### RNA sequencing

The RNA quality was assessed using Bioanalyzer 2100 (Agilent Technologies, Santa Clara, CA, USA). Libraries for cDNA sequencing were constructed using the Illumina TruSeq v2 kit (Illumina Inc., San Diego, CA, USA) according to the manufacturer’s protocol. RNA sequencing of three biological replicates of *E. okinawaensis* midgut samples was performed using HiSeq 2500.

### Functional annotation pipeline

Trinity software (v2.5.1) was used to construct de novo transcriptomes [10], and TransDecoder (v5.2.0) was used to find coding regions within transcripts [11]. The transcriptome sequences were compared through successive execution of BLASTP program (v2.7.1 +) [12] against protein datasets described below.

### Meta-analysis of public data

We utilized transcriptome assemblies available in Transcriptome Shotgun Assembly (TSA) database. If unavailable in TSA, transcriptomes were constructed locally using Trinity. A program (align_and_estimate_abundance.pl) in Trinity software with Kallisto (v0.43.1) was used to estimate the abundance of reads [13].

### Gene enrichment and pathway analyses

Metascape was used for the gene set enrichment analysis [14]. Using EC numbers from our functional annotation pipeline, those genes related to carbohydrate metabolic processes in *E. okinawaensis* were mapped to reference pathway maps in Kyoto Encyclopedia of Genes and Genomes (KEGG) [15].

### Data visualization

A heatmap of the hierarchical clustering and a scatter plot with gene IDs were generated using TIBCO Spotfire Desktop version 7.6.0 (TIBCO Software Inc., Palo Alto, CA, USA) with TIBCO Software’s “Better World” program license.

### Analysis of enolase-coding sequences

HMMsearch program (v3.2.1) [16] was used to detect enolase candidates using profiles of enolase N-terminal domain (Enolase_N, PF03952) and C-terminal domain (Enolase_C, PF00113) in Pfam database (v32.0) [17].

### qRT-PCR

We used 0.5 μg of total RNA purified from the midgut of male and female adults of flight (*S. sipylus*) and flightless (*E. okinawaensis*) stick insects for cDNA synthesis. cDNA synthesis was performed using a PrimeScript™ First-strand cDNA Synthesis Kit (Takara Co. Ltd., Tokyo, Japan) according to the manufacturer’s instructions. qRT-PCR was performed with a Step One plus Real-Time PCR System (Applied Biosystems, Foster City, CA, USA) using the delta-delta Ct method. The 20-μL reaction volumes consisted of 0.5 μL of the cDNA template and primers (Additional file 1: Table S1), and KAPA SYBR Fast qRT-PCR Kit (Nippon Genetics Co. Ltd., Tokyo, Japan) was used according to the manufacturer’s instructions. The *S. sipylus* and *E. okinawaensis* Rp49 sequences were used as endogenous references to standardize the RNA expression levels. All the data were calibrated against universal reference data, and relative quantification (RQ) values of three biological replicates were used to represent the relative expression level against a reference sample.

## Results and discussion

**Fig. 1:**
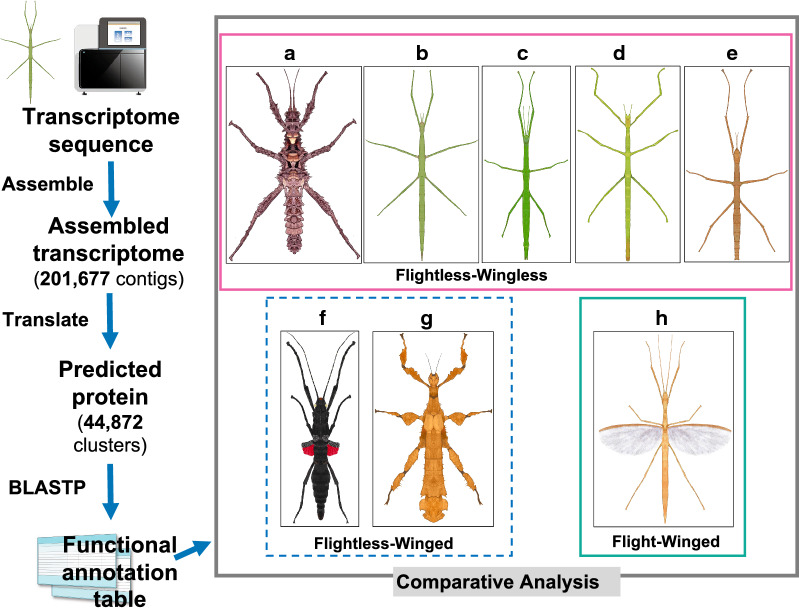
Functional annotation pipeline for *E. okinawaensis* midgut transcriptome assembly. To analyze the *E. okinawaensis* midgut transcriptome, we annotated the translated peptide sequence set through sequential BLASTP using several model organisms and *Manduca sexta* protein sequences, and we then also annotated the translated peptide sequence set via sequential BLASTP using 71 species of protein sequences obtained from the Ensembl Metazoa database. A functional gene annotation pipeline was used for the comparative pathway and gene enrichment analyses of eight species of stick insects using KEGG or Metascape. The stick insect species are as follows: **a** *Aretaon asperrimus* (male); **b** *Entoria okinawaensis* (female); **c** *Clitarchus hookeri* (female); **d** *Ramulus artemis* (female); **e** *Medauroidea extradentata* (female); **f** *Peruphasma schultei* (female); **g** *Extatosoma tiaratum* (female); and **h** *Sipyloidea sipylus* (female). The sequencer and text image drawings are from TogoTV (^©^2016 DBCLS TogoTV/CC-BY-4.0). Mr. Satoshi Goto from the Tabunoki laboratory gifted all image drawings of stick insects

In this study, we developed functional annotation pipeline for *E. okinawaensis* midgut transcriptome (Fig. 1). First, we generated more than 100 million 100-bp paired-end reads from each of three biological replicate RNA libraries of the *E. okinawaensis* midgut (Additional file 1: Table S2). De novo assembly using Trinity [10] produced 201,677 transcripts. After translated to 44,872 protein sequences, a systematic sequence similarity analysis was performed against protein sequence sets of functionally well-annotated organisms (1; human → 2; mouse → 3; *C. elegans* → 4; *D. melanogaster*) in the public database using BLASTP [12]. The organisms used were limited to ensure the use of computationally tractable annotations (Gene Ontology (GO) annotation and others) in Ensembl database (v.93) [18]. In this process, 27,139 proteins were functionally annotated (60.5% of the total predicted peptides). Furthermore, 71 protein sequence sets from Ensembl Metazoa (v40) and of *Manduca sexta* (taken from FTP site at Kansas State University) were added to the reference database for BLASTP search. Ultimately, 35,186 proteins in the transcriptome (78.4%) could be assigned to some gene in the reference dataset. Because most transcripts can be functionally annotated from the human genome, we surmised that most stick insect genes can be annotated from functional information from the human genome.

**Fig. 2:**
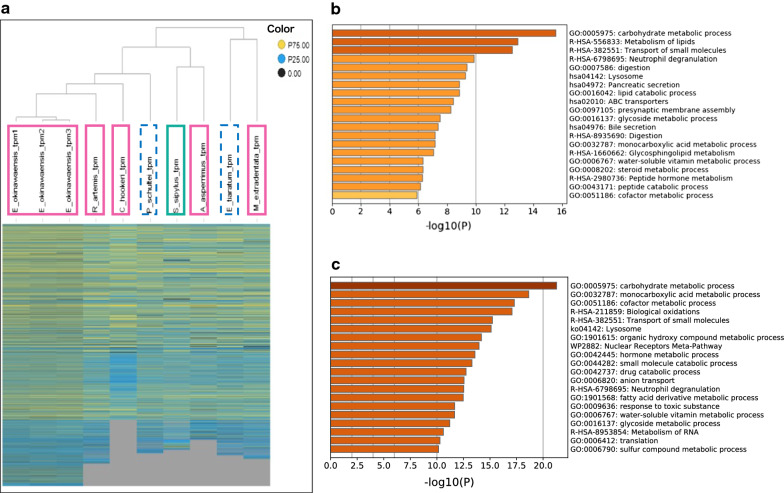
Hierarchical clustering and Gene set enrichment analysis using Metascape of eight stick insect midgut transcriptomes. **a** Hierarchical clustering was performed using TIBCO Spotfire Desktop version 7.6.0. The heatmap is colored based on quartiles. In other words, the gene expression value was sorted in ascending order for each stick insect transcriptome. The first quartile was assigned the middle number between the smallest number and the median of the dataset (blue), and the third quartile was assigned the middle value between the median and the highest value of the dataset (yellow). Genes that did not correspond to *E. okinawaensis* are shown in gray as missing values. The pink-colored solid rectangles show the flightless-wingless species, the green-colored solid rectangles show the flight-winged species, and blue-colored dotted rectangles show the flightless-winged stick insect species. **b** Comparison between flight and flightless insects. **c** Comparison between winged and wingless insects. A bar graph of the enriched terms across genes with high expression in the flight or winged group is shown. The different color intensities indicate significance for the corresponding GO term

To explore stick insect-specific genes and perform a meta-analysis, we collected seven stick insect midgut transcriptome reads and assemblies from public databases. Including our data (*E. okinawaensis*), a total of eight stick insect midgut transcriptomes were analyzed (Additional file 1: Table S3). A comparative gene table with expression abundance information was generated for use in the functional annotation of possible genes, and the hierarchical clustering of eight stick insect midgut transcriptomes was performed (Fig. 2a). *E. okinawaensis* was located in the flightless-wingless cluster, whereas *M. extradentata* was distributed in a different cluster that contained flightless-winged stick insects (Fig. 2a). The corresponding dendrogram indicates that the use of gene expression profiles mainly clustered stick insects with similar characteristics. The availability of RNA-seq reads from the midgut enabled us to perform a whole transcriptome comparison of the eight stick insects selected in this study.

We extracted genes with transcript per million (TPM) values higher than 2-fold for the subsequent gene set enrichment analyses. The examination of the differentially expressed genes in the flight and winged stick insects showed that the expression of 427 unique genes was elevated in the flight group, whereas the expression of 2636 unique genes was downregulated in the flight group. Enrichment analysis by Metascape [14] resulted in the generation of two genetic functional groups between flight and flightless, and revealed that carbohydrate metabolic process (GO:0005975) and metabolism of lipids (R-HSA-556833) were significantly upregulated in the flight group (Fig. 2b). Enrichment analysis of the upregulated genes in winged stick insects also showed significant functional enrichment of genes annotated with carbohydrate metabolic processes (Fig. 2c). These findings indicate that carbohydrate metabolic processes are shared between flight and winged stick insect groups.

The pathway diagram visualization of carbohydrate metabolic processes in which enzyme-coding genes in the *E. okinawaensis* midgut transcriptome were marked indicated that the transcripts for enzymatic genes involved in glycolysis were fully reconstructed from the midgut transcriptome sequencing data using KEGG database [15] (Additional file 3: Fig. S1). Additionally, we compared the conservation of carbohydrate metabolic processes among the eight stick insect species and found that carbohydrate metabolic processes were well conserved among these species (Additional file 2: Table S4, excel file). We estimate that different host plants would affect the expression profile of transcripts involved in the carbohydrate metabolic process in the insect midgut because the amount of nutrients depends on the condition and development of their host plants [19, 20]. Although the eight stick insects have different host plants, they can also eat a wide range of plants [9, 21–25].

**Fig. 3:**
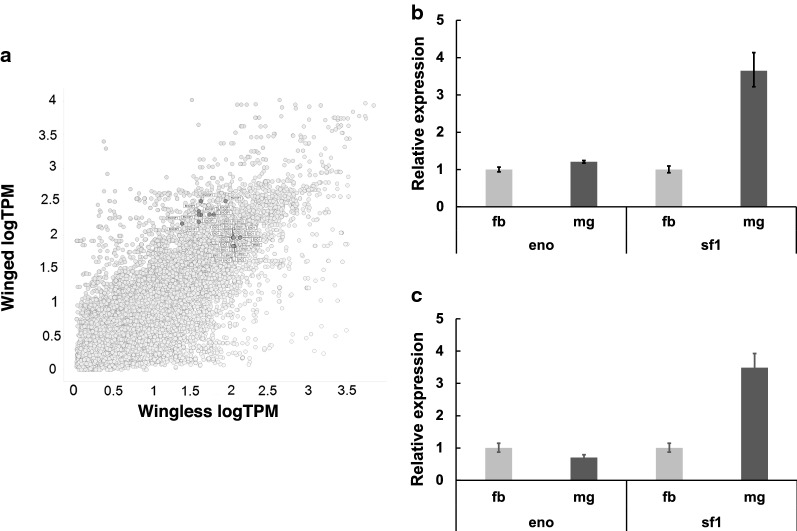
Comparison of the winged and wingless groups. **a** Differentially expressed transcripts are plotted based on the logarithm-transformed TPM (log TPM) values on the graph. The X-axis shows the wingless group, and the Y-axis shows the winged group. The gray-colored dots indicate transcripts with more than 2-fold differential expression in the comparison of the winged and wingless stick insects. The blue dots indicate significantly differentially expressed transcripts related to carbohydrate metabolic processes identified from the comparison of the winged and wingless groups. **b** Enolase and ENOSF1 mRNA expression in *S. sipylus* (flight-winged stick insect). **c** Enolase and ENOSF1 mRNA expression in *E. okinawaensis* (flightless-wingless stick insect). The relative mRNA expression levels in the fat body and midgut are presented as relative quantification (RQ) values. The RQ values show the relative expression levels calculated based on an expression value in the fat body equal to 1. The error bars represent the relative minimum/maximum expression levels of the mean RQ values. Rp49 was used as the endogenous control. Triplicate technical replicates were included in the study. *eno* enolase, *sf1* ENOSF1, *fb* fat body, *mg* midgut

Among the highly expressed genes in carbohydrate metabolic processes, we found enolases in the winged and wingless stick insect groups (Fig. 3a). After a careful investigation of domain structures, this group of transcripts corresponded with a stick insect homolog of enolase superfamily 1 (ENOSF1), which plays a role in the catabolism of L-fucose (UniProt: Q7L5Y1). Thus, the gene expression levels of enolase and ENOSF1 were compared among the eight stick insect species based on TPM value, and we found that the expression of the ENOSF1 gene in the winged stick insects was relatively higher than that in the wingless stick insects (Additional file 1: Table S5). We then compared the expression of the ENOSF1 gene between the midgut and fat body by qRT-PCR, and in winged and wingless stick insects, the expression of the ENOSF1 transcript in the midgut was higher than that in the fat body (Fig. 3b, c). Enolase is an important glycolytic enzyme in organisms, and ENOSF1 is related to the production of energy from L-fucose in mitochondria. Thus, enolase and ENOSF1 play a role in the production of energy in the stick insect midgut, similar to the findings from other organisms, and ENOSF1 may be related to the evolutionary process between wingless and flying stick insects.

It is known that the flight fuel differs depending on the type of insect. The transition from rest to flight in many insects is accompanied by a 100-fold increase in the metabolic rate [26]. Therefore, sufficient enzymatic activity is needed for the production of energy in flight [27]. Short-distance-travel insects use carbohydrates as their main energy source [26]. The preferred energy source of long-distance-travel insects is carbohydrates, and these insects then change their energy source from carbohydrates to lipids [28]. In most insects, carbohydrates are used as the main energy source because carbohydrates are hydrophilic substances and move faster than lipids into insect bodies. Therefore, our findings might point to some gene expression bias in stick insects with evolutionary flight ability.

## Limitations

- The functional annotation pipeline cannot detect stick insect-specific genes. Hence, further comparative sequence analyses are needed to investigate stick insect-specific genes.
- The environmental conditions in their habitat also affect the metabolic system of insects [29], but we did not find this environmental condition-related effect.

## Supplementary Information

**Additional file 1: Table S1**. qRT-PCR primers used in this study. **Table S2**. Total numbers and read bases and with corresponding IDs in public databases. **Table S3**. Characteristics of the stick insects investigated in this study. **Table S5**. Representative TPM values for enolase and ENOSF1 in each stick insect.

**Additional file 2: Table S4.** Reconstructed glycolysis/gluconeogenesis pathway from eight stick insect species based on the KEGG pathway database.

**Additional file 3: Fig. S1.** Reconstructed glycolysis/gluconeogenesis pathway from the KEGG metabolic pathway database. Carbohydrate metabolic process-related genes were mapped to the KEGG reference pathway diagram for glycolysis/gluconeogenesis, and enzyme-coding genes assigned to the midgut transcriptome of *E. okinawaensis* are colored red.

## Data Availability

The RNA sequencing reads reported in this article are available in the Sequence Read Archive (SRA) under the accession ID DRA007226. The assembled transcriptome sequences are available in the Transcriptome Shotgun Assembly (TSA) database under the accession ID IADO01, and the estimated abundance of transcripts is available from the Genomic Expression Archive (GEA) under the accession ID E-GEAD-295.
