# Supplementary material for: De novo transcriptome analysis for examination of the nutrition metabolic system related to the evolutionary process through which stick insects gain the ability of flight (Phasmatodea)
Source: BMC Res Notes. 2021 May 13;14:182. doi: 10.1186/s13104-021-05600-0 (PMC8120901; doi:10.1186/s13104-021-05600-0)
Supplement: Supplementary file 1 — Additional file 1: Table S1. qRT-PCR primers used in this study. Table S2. Total numbers and read bases and with corresponding IDs in public databases. Table S3. Characteristics of the stick insects investigated in this study. Table S5. Representative TPM values for enolase and ENOSF1 in each stick insect. [file 13104_2021_5600_MOESM1_ESM.docx]

**Title**: *De novo* transcriptome analysis for examination of the nutrition metabolic system related to the evolutionary process through which stick insects gain the ability of flight (Phasmatodea).

**Authors**

Takuma Sakamoto^1,2^, Shunya Sasaki^2^, Nobuki Yamaguchi^2^, Miho Nakano^2^, Hiroki Sato^2^, Kikuo Iwabuchi^2^, Hiroko Tabunoki^1,2^, Richard J. Simpson^1,3^, and Hidemasa Bono^4,5^*

**Affiliations**

^1^Institute of Global Innovation Research, Tokyo University of Agriculture and Technology, 3-5-8 Saiwai-cho, Fuchu, Tokyo, 183-8509, Japan.

^2^Department of Science of Biological Production, Graduate School of Agriculture, Tokyo University of Agriculture and Technology, Tokyo, Japan.

^3^Department of Biochemistry and Genetics, La Trobe Institute for Molecular Science (LIMS), La Trobe University, Melbourne, Victoria, 3086, Australia.

^4^Database Center for Life Science (DBCLS), Joint Support-Center for Data Science Research, Research Organization of Information and Systems (ROIS), Mishima, Shizuoka, Japan.

^5^Program of Biomedical Science, Graduate School of Integrated Sciences for Life, Hiroshima University, Hiroshima, Japan.

*Corresponding author: Hidemasa Bono [bonohu@hiroshima-u.ac.jp](mailto:bonohu@hiroshima-u.ac.jp)

**Supplementary information**

**Table S1. qRT-PCR primers used in this study**

| **Primer** | **Forward** | **Reverse** |
| --- | --- | --- |
| *S. sipylus* Eno | 5’-AGCCAAAGAGAATGGGTGGG-3’ | 5’-CGCAGATCCACCCAACTCTT-3’ |
| *S. sipylus* Eno_sf1 | 5’- ATCCAGCATACACCACGCAA-3’ | 5’-AGCCGATCACCTCTCTGACT-3’ |
| *S. sipylus* Rp49 | 5’-GTGCTGAAGTTACGGACGGA-3’ | 5’-AAGCGACGACGAACTCTGTT-3’ |
| *E. okinawaensis* Eno | 5’-GCACCATGGTGTCTCACAGA-3’ | 5’- TTCTTGCCAGCAAACTTCGC-3’ |
| *E. okinawaensis* Eno_sf1 | 5’-CCACTCTGGAAGTTGCTCGT-3’ | 5’-TGGATAGCCTGTCACCCTGA-3’ |
| *E. okinawaensis* Rp49 | 5’-GGTCTTGGTGTCCTGGTTGT-3’ | 5’-GTAAACCGGACGTATGGCCA-3’ |

**Table S2. Total numbers and read bases and with corresponding IDs in public databases**

| **Biosample ID** | **SRA Experiment ID** | **SRA Run ID** | **Numbers of reads** | **Read bases (bp)** |
| --- | --- | --- | --- | --- |
| SAMD00135006 | DRX138869 | DRR148118 | 120,866,442 | 12,207,510,642 |
| SAMD00135007 | DRX138870 | DRR148119 | 124,763,020 | 12,601,065,020 |
| SAMD00135008 | DRX138871 | DRR148120 | 128,518,618 | 12,980,380,418 |

**Table S3. Characteristics of the stick insects investigated in this study**

| **Species** | **Sex** | **Flight ability** | **Wing** | **TSA ID** |
| --- | --- | --- | --- | --- |
| *Peruphasma schultei* | Not reported | No | Yes | Not available |
| *Sipyloidea sipylus* | Not reported | Yes | Yes | GAWF01 |
| *Extatosoma tiaratum* | Female | No | Yes | GAWG01 |
| *Medauroidea extradentata* | Female | No | No | GAWD01 |
| *Aretaon asperrimus* | Not reported | No | No | GAWC01 |
| *Entoria okinawaensis* | Male and Female | No | No | IADO01 |
| *Clitarchus hookeri* | Female | No | No | Not available |
| *Ramulus artemis* | Female | No | No | GAWE01 |

**Table S4. Reconstructed glycolysis/gluconeogenesis pathway from eight stick insect species based on the KEGG pathway database**

**Table S5. Representative TPM values for enolase and ENOSF1 in each stick insect**

| **Species** | **Enolase** | **ENOSF1** | **Flying ability/wing** |
| --- | --- | --- | --- |
| *Peruphasma schultei* | 73.4 | 156.5 | No/Yes |
| *Sipyloidea sipylus* | 105.3 | 384.7 | Yes/Yes |
| *Extatosoma tiaratum* | 99.5 | 451.6 | No/Yes |
| *Medauroidea extradentata* | 92.2 | 81.0 | No/No |
| *Aretaon asperrimus* | 269.3 | 43.3 | No/No |
| *Entoria okinawaensis* | 53.7 | 105.0 | No/No |
| *Clitarchus hookeri* | 270.3 | 28.1 | No/No |
| *Ramulus artemis* | 77.6 | 116.2 | No/No |
