# Supplementary figures and images for: De novo transcriptome analysis for examination of the nutrition metabolic system related to the evolutionary process through which stick insects gain the ability of flight (Phasmatodea)
Source: BMC Res Notes. 2021 May 13;14:182. doi: 10.1186/s13104-021-05600-0 (PMC8120901; doi:10.1186/s13104-021-05600-0)

# GLYCOLYSIS / GLUCONEOGENESIS

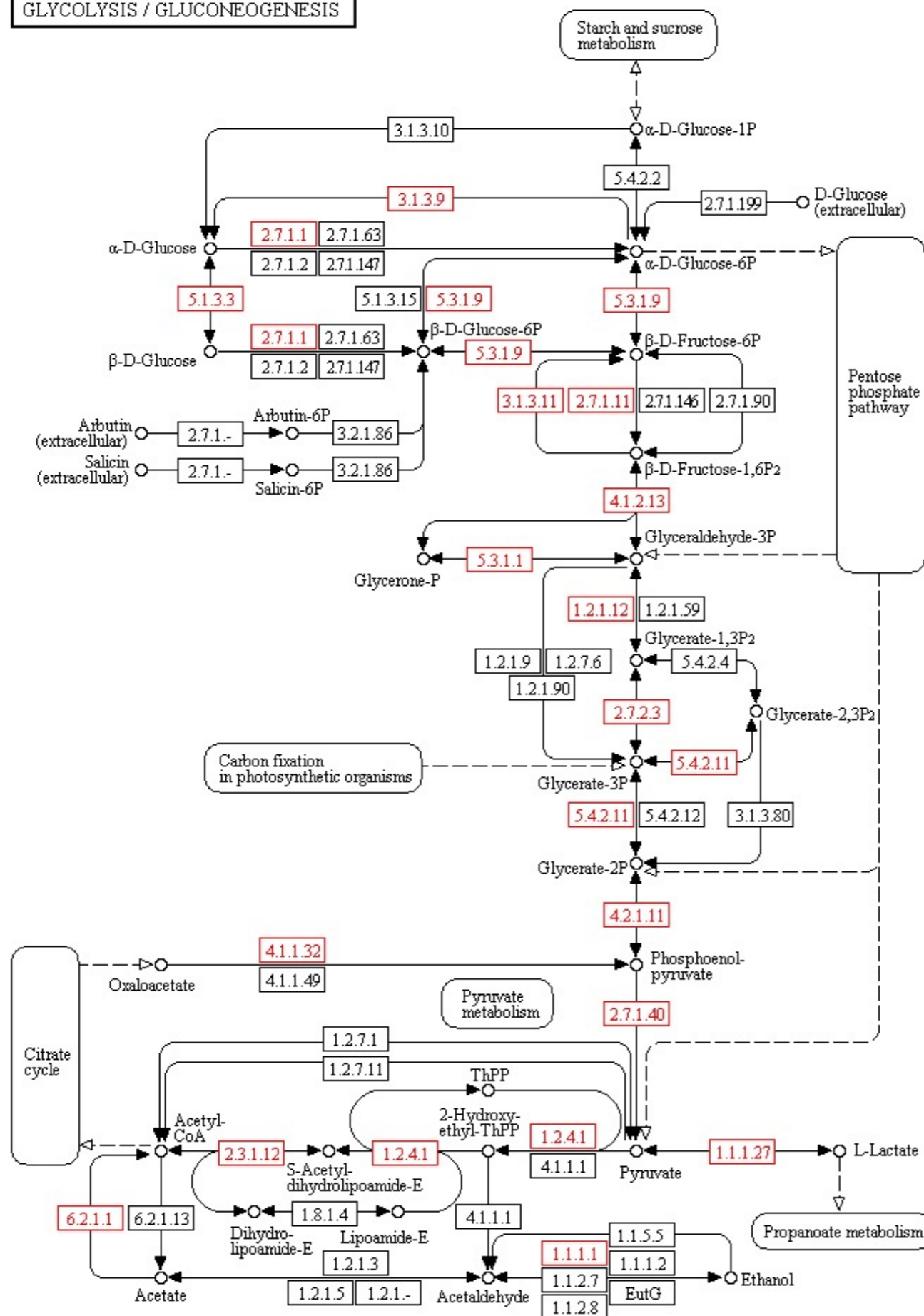

Fig. S1

Supplement: Supplementary file 3 — Additional file 3: Fig. S1. Reconstructed glycolysis/gluconeogenesis pathway from the KEGG metabolic pathway database. Carbohydrate metabolic process-related genes were mapped to the KEGG reference pathway diagram for glycolysis/gluconeogenesis, and enzyme-coding genes assigned to the midgut transcriptome of E. okinawaensis are colored red. [file 13104_2021_5600_MOESM3_ESM.pdf]
